# The effects of heat and hydrogen peroxide treatment on the osteoinductivity of demineralized cortical bone: a potential method for preparing tendon/ligament repair scaffolds

**DOI:** 10.1093/rb/rbae116

**Published:** 2024-09-25

**Authors:** Shukun He, Ruonan Hu, Xuan Yao, Jing Cui, Huimin Liu, Min Zhu, Liangju Ning

**Affiliations:** Department of Orthopedic Surgery and Orthopedic Research Institute, Stem Cell and Tissue Engineering Research Center, State Key Laboratory of Biotherapy, West China Hospital, Sichuan University, Chengdu, 610041, China; Department of Orthopedics, First Affiliated Hospital, College of Medicine, Zhejiang University, Hangzhou, 310058, China; Department of Orthopedic Surgery and Orthopedic Research Institute, Stem Cell and Tissue Engineering Research Center, State Key Laboratory of Biotherapy, West China Hospital, Sichuan University, Chengdu, 610041, China; Department of Clinical Hematology, Faculty of Laboratory Medicine, Army Medical University, Chongqing, 400038, China; Department of Orthopedic Surgery and Orthopedic Research Institute, Stem Cell and Tissue Engineering Research Center, State Key Laboratory of Biotherapy, West China Hospital, Sichuan University, Chengdu, 610041, China; Department of Orthopedic Surgery and Orthopedic Research Institute, Stem Cell and Tissue Engineering Research Center, State Key Laboratory of Biotherapy, West China Hospital, Sichuan University, Chengdu, 610041, China; Department of Orthopedic Surgery and Orthopedic Research Institute, Stem Cell and Tissue Engineering Research Center, State Key Laboratory of Biotherapy, West China Hospital, Sichuan University, Chengdu, 610041, China; Department of Orthopedic Surgery and Orthopedic Research Institute, Stem Cell and Tissue Engineering Research Center, State Key Laboratory of Biotherapy, West China Hospital, Sichuan University, Chengdu, 610041, China

**Keywords:** demineralized cortical bone, heat treatment, hydrogen peroxide treatment, cell differentiation, osteoinductivity

## Abstract

Recent studies have indicated that demineralized cortical bone (DCB) may be used to repair tendons and ligaments, such as the patellar tendon and anterior cruciate ligament (ACL). Hydrogen peroxide (H_2_O_2_) has been shown to reduce the osteoinductivity of DCB, and heat treatment may also decrease the osteoinductivity of DCB. The purpose of this study was (i) to determine whether heat treatment reduces the osteoinductivity of DCB and (ii) to compare the effectiveness of heat treatment and H_2_O_2_ treatment on BMP-2 inactivation. DCB was prepared by immersion in 0.6 N hydrochloric acid, and DCB-H and DCB-HO were prepared by heat treatment (70°C for 8 h) and H_2_O_2_ treatment (3% H_2_O_2_ for 8 h), respectively. The surface topographies, elemental distributions and histological structures of the scaffolds were observed by scanning electron microscopy (SEM), Fourier transform infrared spectroscopy (FT-IR) and histological staining. The viability and osteogenic differentiation of TDSCs cultured on the scaffolds were evaluated *via* live/dead cell staining and Cell Counting Kit-8 (CCK-8) testing, real-time polymerase chain reaction (RT-PCR) and western bolt (WB) analysis, alkaline phosphatase activity (ALP) and alizarin red S (ARS) staining. The intramuscular implantation of the scaffolds in rats was also used to evaluate the effect of heat treatment and H_2_O_2_ treatment on the osteoinductivity of DCB. Our results demonstrated that both treatments removed BMP-2 and osteocalcin (OCN) within the DCB and that DCB-H and DCB-HO had good cytocompatibility and reduced the osteogenic differentiation of TDSCs. Moreover, the *in vivo* results indicated that the DCB-H and DCB-HO groups had smaller areas of osteoid formation than did the DCB group, and the DCB-HO group had the smallest area among the three groups. Our study demonstrated that heat treatment could reduce the osteoinductivity of DCB, and that H_2_O_2_ treatment was more effective than heat treatment.

## Graphical abstract

**Graphical Abstract:**
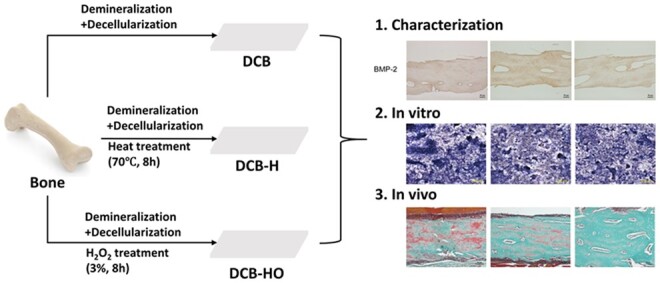

## Introduction

Tendon injury, including injury to the rotator cuff, Achilles tendons and patellar tendons, is one of the most frequently occurring diseases [1–3]. Unfortunately, the intrinsic healing process of tendon injuries is slow, primarily due to the inherent characteristics of tendons, including low innervation, hypovascularity and hypocellularity, which collectively impede their capacity for effective repair [4–6]. At present, surgical treatments for tendon injuries include direct suturing, autografting and allografting. Autografts and allografts are often used for tendon injuries that cannot be sutured directly. However, autografts have limitations such as limited graft materials and complications at donor sites [7]. Similarly, allografts suffer from drawbacks, including infection risk and immune rejection [8, 9]. To overcome these disadvantages, various biomaterials from animals (such as decellularized dermal matrix and acellular small intestine mucosa) have been fabricated to repair or regenerate injured tendon or ligament tissues [10].

Demineralized bone matrix (DBM) has been used for bone tissue engineering because of its good biocompatibility, biomechanics and osteoinductivity [11]. DBM is a scaffold consisting of collagen (mainly type I with some types IV and X) and several growth factors (such as bone morphogenic proteins [BMPs]) [12]. The remaining collagen structure of DBM could provide a 3D microenvironment for the ingrowth of host stem cells and vascular tissue, while growth factors, including BMP, could be released from DBM and exert their osteoinductive potential [13]. Recently, DBM has shown to be suitable for tissue engineering of tendons and ligaments [14–18]. Jackson *et al.* [14] first used DBM to reconstruct the anterior cruciate ligament (ACL) in goats and reported that the intra-articular portion of the graft was transformed into a ligament-like structure. Yamada [15] prepared a bone-demineralized bone–bone scaffold to mimic the structure of the bone–ligament–bone to reconstruct the ACL and medial collateral ligament (MCL) in rats, and reported that the appearance of the scaffold was transformed into white ligament-like tissue at 8 weeks after surgery. Sundar *et al.* [16] reported that DBM improved the healing of the tendon–bone interface of the ovine patellar tendon, and that the interposition region of DBM was completely remodeled. Elnikety *et al.* [17] also used demineralized cortical bone (DCB) to repair ovine patellar tendon defects, and the results revealed that the characteristic crimp of the normal tendon was found in the DCB. Dickerson *et al.* [18] prepared a continuous demineralized bone–bone scaffold to repair the tendon–bone interface in sheep, and reported that more organized and aligned collagen fibers were present in the scaffold-treated samples. These studies demonstrated the capacity of DBM/DCB for the reconstruction of tendons and ligaments, possibly because of the similar composition of collagen (type I) and the physiological relationship between bone and tendon/ligament.

The biological factors of DBM/DCB could provide a considerable advantage in bone regeneration; however, they may lead to adverse reactions (such as heterotopic ossification) in soft tissue regeneration applications [19, 20]. Heterotopic ossification is always associated with pain, limited mobility and weakened biomechanics of tendons/ligaments [21, 22]. Therefore, removing the inherent osteoinductivity of DBM/DCB to eliminate the risk of heterotopic ossification is important for soft tissue repair applications. Previous studies have indicated that BMP-2, a biological factor in DBM/DCB, can promote the osteogenic differentiation of cells and the formation of new bone, which is thought to be involved in the formation of heterotopic ossification [23–25]. To remove BMP-2 from DBMs, some studies have focused on hydrogen peroxide (H_2_O_2_) [26, 27]. DePaula *et al.* [26] reported that 3% H_2_O_2_ could reduce the osteoinductivity of DBMs when DBMs were implanted into the hamstring muscle of nude mice. Carpenter *et al.* [27] also demonstrated that the osteoinductivity of DBMs implanted in rats decreased after long-term incubation with 3% H_2_O_2_. In addition to chemical methods, some researchers have used physical methods (such as heat treatment) to inactivate BMP-2. Yano *et al.* [28] reported that the bone-inducing capacity of BMP-2 decreased after heating at 70°C for 8 h or 90°C for 2 h. However, the use of heat treatment to reduce the osteoinductivity of DCBs has still not been investigated. In this study, first, we aimed to determine whether heat treatment reduces the osteoinductivity of DCBs through osteogenic differentiation of stem cells and intramuscular implantation in rats. Second, we compared the effectiveness of heat treatment and H_2_O_2_ treatment on the basis of BMP-2 inactivation.

## Materials and methods

### Preparation and characterization

All experiments were performed in accordance with standard guidelines approved by the Sichuan University Animal Care and Use Committee (No. 2019151A). DCB was prepared according to our published protocol [29, 30]. In brief, soft tissues and cancellous bones were removed from bovine bones, and cortical bone cylinders were made with an automatic cutting machine (Q-80Z, Weiran, China). Then, the bone cylinders were immersed in 0.6 N hydrochloric acid (HCl, Chron Chemicals, China) at room temperature for complete demineralization according to published methods [31, 32]. After demineralization for 48 h, bone cylinders were manufactured into bone slices (10 mm × 5 mm × 0.3 mm) *via* a freezing microtome (CM1950, Leica, Germany) along the long axis. Next, the bone slices were degreased with a mixed solution of methanol and ethanol (Chron Chemicals, China, *V*/*V* = 1:1), and then the samples were decellularized with 0.5% Triton X-100 (Amresco, USA), 150 IU/ml DNase (Roche, Germany) and 100 μg/ml RNase (Roche, Germany). After being rinsed in phosphate-buffered saline (PBS) for 24 h, the DCB was collected. Heat treatment and H_2_O_2_ treatment were used to reduce the osteoinductivity of DCB according to published studies [26, 28]. DCB-H was prepared by heat treatment at 70°C for 8 h, and DCB-HO was prepared through immersion in 3% H_2_O_2_ for 8 h at room temperature. Finally, DCB, DCB-H and DCB-HO were rinsed in PBS overnight and lyophilized for the next step.

The surface morphologies of DCB, DCB-H and DCB-HO were characterized by scanning electron microscopy (SEM, EVO 10, Carl Zeiss, Germany). Fourier transform infrared spectroscopy (FT-IR, Nicolet 6700, Thermo Scientific, USA) was used to detect the degree of demineralization in these three types of scaffolds. In addition, all the scaffolds were observed *via* hematoxylin and eosin (H&E) staining, Masson's trichrome staining and immunohistochemical (IHC) staining for BMP-2 (Bioss, USA) and osteocalcin (OCN, Bioss, USA). Semiquantitative analyses of BMP-2- and OCN-positive cells were performed at a total magnification of 200× (five random images per sample, *n* = 3 per group) *via* ImageJ software (NIH, USA) with the IHC toolbox plugin [33].

### Cytotoxicity assays

Tendon-derived stem cells (TDSCs) from Sprague–Dawley (SD) rats were isolated and cultured according to our published methods [34]. TDSCs were isolated from Achilles tendons and cultured in low-glucose Dulbecco’s modified Eagle’s medium (Gibco, USA) supplemented with 20% fetal bovine serum (Gibco, USA) and 1% penicillin/streptomycin (Gibco, USA). The growth medium was changed every 2–3 days, and all experiments were performed with TDSCs at passage 3. DCB, DCB-H and DCB-HO were added to 24-well plates (Corning, USA), and TDSCs were seeded on the scaffolds at a density of 5 × 10^3^ cells/cm^2^ in growth medium. After a 24-h incubation period, a live/dead assay kit (Proteintech, USA) was used to assess the cytotoxicity of the scaffolds. All the scaffolds were stained with calcein-AM and propidium iodide (PI) for 30 min, and then the scaffolds were observed *via* fluorescence microscopy (Axio Imager Z2, Zeiss, Germany). Moreover, DCB, DCB-H and DCB-HO were incubated in standard DMEM at a concentration of 3 cm^2^/ml at 37°C for 24 h. The supernatant was subsequently collected to serve as an extract for further cytotoxicity assays. TDSCs were seeded in 96-well cell culture plates with growth medium at a concentration of 2 × 10^3^ cells/well. After a 24-h incubation period, the medium was removed and replaced with either standard DMEM or 100% extracts. The proliferation of TDSCs cultured in extracts from the different scaffolds was quantitatively assessed via a Cell Counting Kit-8 (CCK-8; Dojindo, Japan) after 1, 4 and 7 days of incubation.

### Real-time polymerase chain reaction (RT-PCR) analysis

To evaluate the effects of heat and hydrogen peroxide treatment of stem cells treated with DCB on osteogenic differentiation, the expression of osteogenic differentiation-specific markers at the mRNA level was examined in TDSCs cultured on the scaffolds in normal media for 7 or 14 days. Total RNA was extracted with TRIzol reagent (Invitrogen, USA), and cDNA was synthesized by reverse transcription using the GoScript Reverse Transcription System (Promega, USA). Rat-specific primers for genes, including glyceraldehyde-3-phosphate dehydrogenase (*GAPDH*), collagen I (*COLI*), runt-related transcription factor 2 (*RUNX2*), alkaline phosphatase (*ALP*), *OCN* and osteopontin (*OPN*), were synthesized by Sango Biotech (Shanghai, China, Table 1). The synthesized cDNA was amplified *via* a LightCycler 96 system (Roche, Germany). The expression of genes was normalized to that of *GAPDH*, and the 2^−△△Ct^ method was used to calculate the relative expression levels of target genes.

**Table 1.** Primer sequences for RT-PCR

Gene	5′–3′	Primer
*GAPDH*	Forward	GCAAGTTCAACGGCACAG
	Reverse	GCCAGTAGACTCCACGACAT
*COLI*	Forward	CGAGTATGGAAGCGAAGG
	Reverse	AGTGATAGGTGATGTTCTGG
*RUNX2*	Forward	CCCAGTATGAGAGTAGGTGTCC
	Reverse	GGGTAAGACTGGTCATAGGACC
*ALP*	Forward	CATCGGACCCTGCCTTAC
	Reverse	GGAGACGCCCATACCATC
*OCN*	Forward	ATTGTGACGAGCTAGCGGAC
	Reverse	TCGAGTCCTGGAGAGTAGCC
*OPN*	Forward	GCACCACTCGCTTCTTTG
	Reverse	TTGTTGATGTCCTGCTCCT

### Western blot analysis

For western blot analysis, the expression of osteogenic differentiation-specific markers at the protein level was examined in TDSCs cultured on the scaffolds in normal media for 7 or 14 days. At the designated time points, total proteins (*n* = 3 for each group) were extracted and quantified. After protein transfer, the PVDF membranes were incubated using the following primary antibodies: β-actin (1:5000, Servicebio, China), ALP (1:1000, Huabio, China), OPN (1:1000, Servicebio, China) overnight at 4°C. Next, the membranes were washed and incubated with a secondary antibody (1:3000, Servicebio, China) for 30 min at 25°C. After washing with Tris-buffered saline containing 0.1% Tween-20, the target protein bands were detected *via* a chemiluminescence imaging system (Servicebio, China), and their intensities were measured with AIWBwell™ software and then normalized to β-actin.

### ALP activity and alizarin red S (ARS) staining

To assess the effects of heat and hydrogen peroxide treatment of DCB on the osteogenesis of stem cells at the protein level, TDSCs (1 × 10^4^ cells/cm^2^) were seeded onto scaffolds and cultured in osteogenic induction medium for a period of 14 days. Then, ALP activity was assayed using a BCIP/NBT alkaline phosphatase color development kit (Beyotime Institute of Biotechnology, China), and calcium deposits were detected by staining with an osteoblast mineralized nodule staining kit (ARS method; Beyotime Institute of Biotechnology, China). ALP-positive cells and calcium deposits were counted under an inverted microscope (ECLIPSE Ti2, Nikon, Japan) in four randomly selected fields.

### Surgical procedure

Eighteen SD rats (male, 200–250 g) were used for intramuscular implantation under the guidance of a published study [35]. After anesthesia, all the animals (*n* = 3 per group) underwent bilateral implantation in the dorsal muscle region. All the rats were evaluated daily for wound infection, and the implants were harvested at 4 and 12 weeks after surgery.

### Histological analysis

All samples were fixed in 4% paraformaldehyde and embedded in paraffin, and 5 μm thick sections were cut for all histological staining. The tissue sections were subjected to H&E, Masson’s trichrome and IHC staining for BMP-2 and OCN. For the observation of osteoid formation, the tissue sections were also subjected to Goldner trichrome staining (Solarbio, China) [36]. Positive staining for BMP-2 and OCN was analysed at a total magnification of 200×, and new osteoid formation was analysed by outlining the area of metachromasia in sections at a total magnification of 200× (five random images per sample, *n* = 3 per group). Images were captured with an upright microscope (AX10 imager A2, Zeiss, Germany), and semiquantitative analyses were carried out with ImageJ.

### Statistical analysis

The data are presented as the mean ± standard deviation (SD). For normally distributed data, one-way analysis of variance (ANOVA) with a *post hoc* least significant difference (LSD) test was used for comparisons among three groups. For nonnormally distributed data, the Kruskal–Wallis *H* test with *post hoc* Dunn–Bonferroni correction was used for comparisons among three groups. A value of *P* < 0.05 was considered to indicate statistical significance, and the statistical analysis was performed with SPSS statistics software (version 26, IBM, USA).

## Results

### Characterization

**Figure 1.**
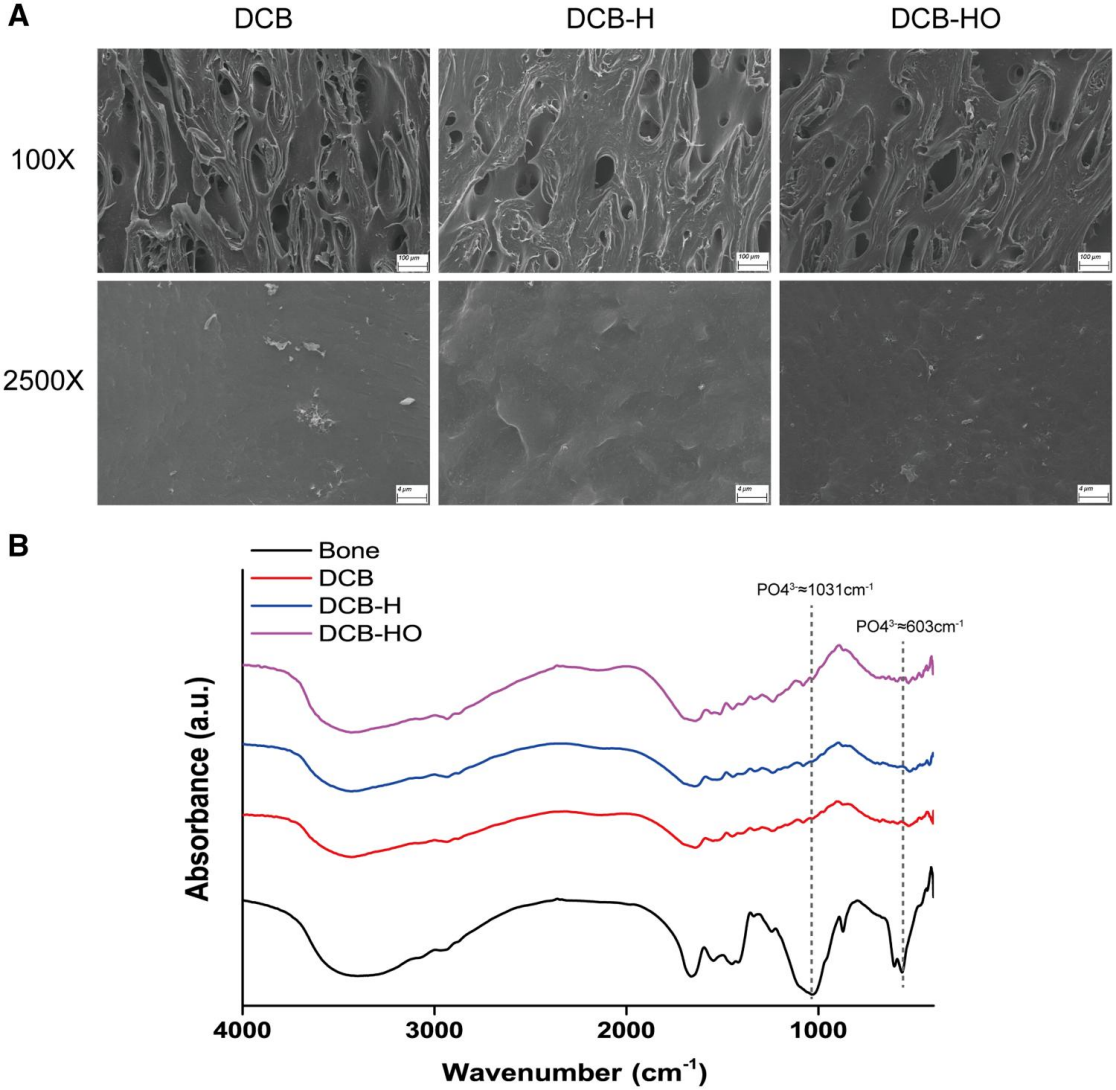
Surface morphological observations and FT-IR spectral analysis of DCB, DCB-H and DCB-HO. (**A**) SEM images. (**B**) FT-IR.

**Figure 2.**
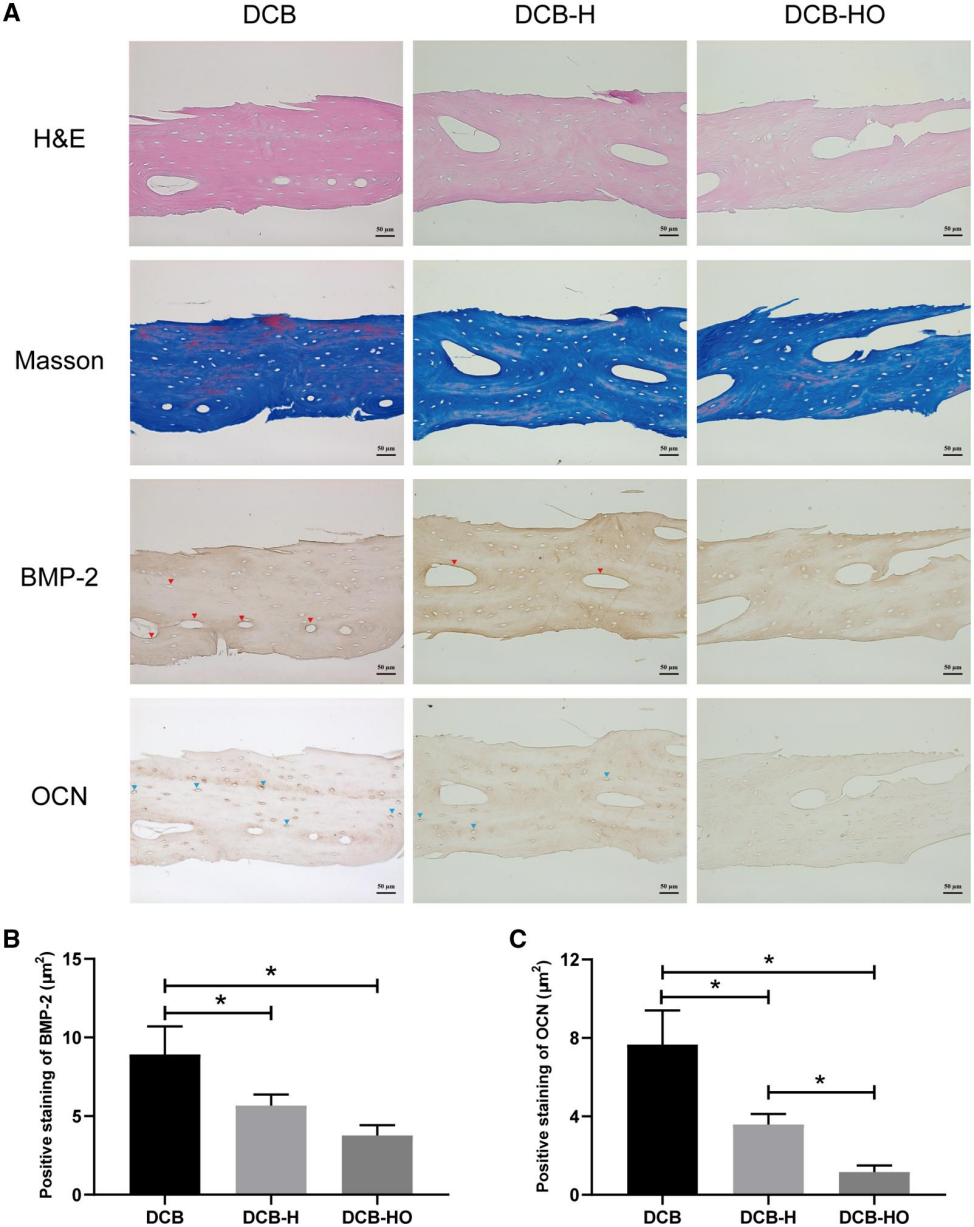
Histomorphology and immunohistochemical analysis of DCB, DCB-H and DCB-HO. (**A**) Representative images of H&E, Masson and IHC staining of the scaffolds. The red arrow indicates positive staining for BMP-2. The blue arrow indicates positive staining for OCN. Scale bar = 50 μm. (**B**) Semiquantitative analysis of BMP-2-positive staining. (C) Semiquantitative analysis of OCN-positive staining. * indicates *P* < 0.05.

SEM revealed that DCB, DCB-H and DCB-HO had similar collagen structures, which indicated that heat treatment and H_2_O_2_ treatment did not change the microstructure of the DCB (Figure 1A). The FT-IR results demonstrated that DCB, DCB-H and DCB-HO were completely demineralized (Figure 1B). After decellularization, H&E and Masson staining revealed that the cells within the DCB, DCB-H and DCB-HO groups were completely removed, and the collagen structure of the scaffolds was well-preserved (Figure 2A). IHC revealed that BMP-2 and OCN could be removed after heat treatment and H_2_O_2_ treatment (BMP-2: DCB vs DCB-H vs DCB-HO = 8.92 ± 1.78 μm^2^ vs 5.67 ± 0.69 μm^2^ vs 3.76 ± 0.65 μm^2^; OCN: DCB vs DCB-H vs DCB-HO = 7.66 ± 1.74 μm^2^ vs 3.59 ± 0.54 μm^2^ vs 1.16 ± 0.34 μm^2^; Figure 2B and C). In addition, H_2_O_2_ treatment was more effective than heat treatment in terms of OCN removal (Figure 2C).

### Cytotoxicity

**Figure 3.**
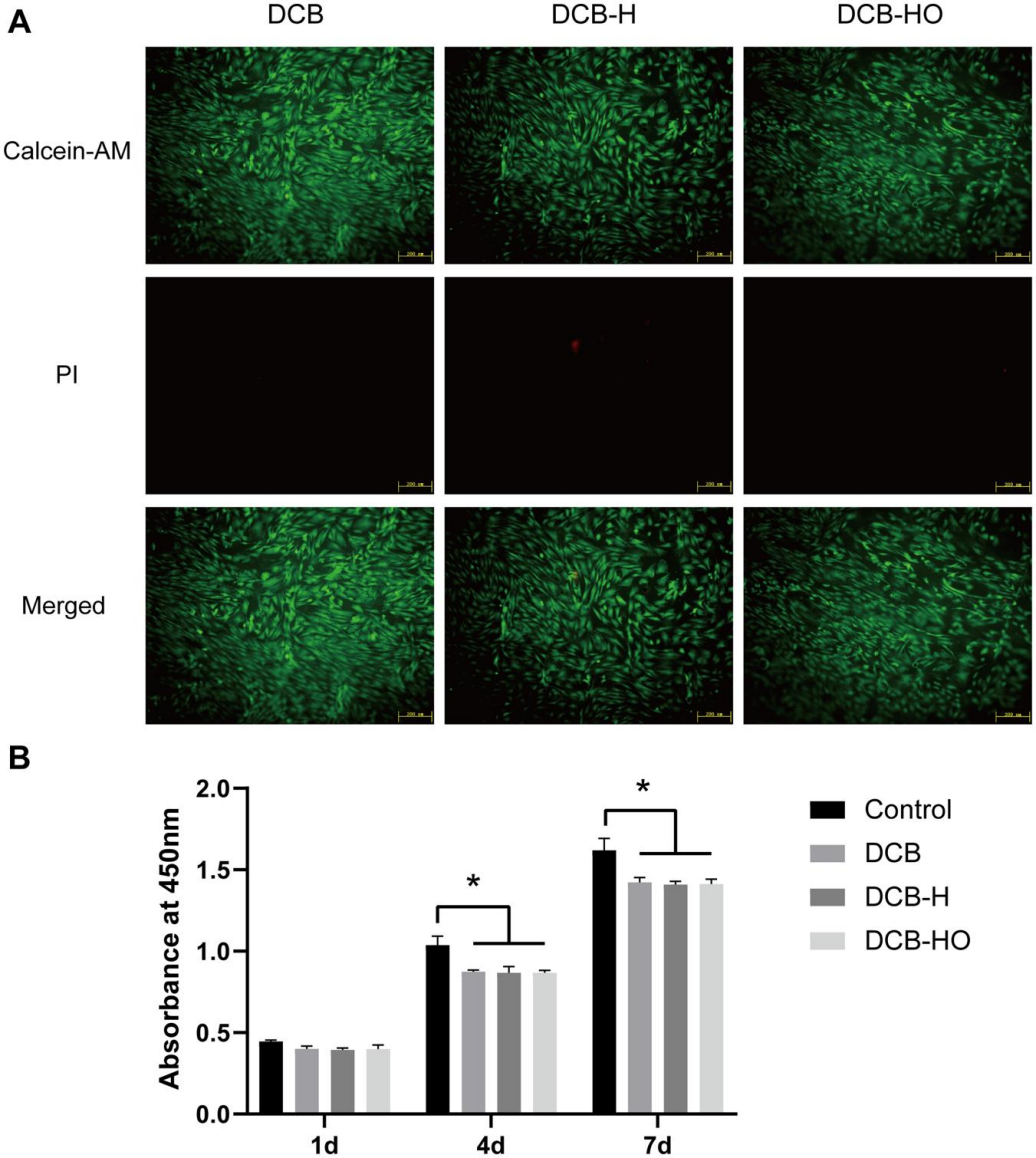
Cytotoxicity analysis of DCB, DCB-H and DCB-HO. (**A**) Live/dead staining of TDSCs seeded on different scaffolds at 24 h by fluorescence microscopy (green, live cells; red, dead cells). Scale bar = 200 μm. (**B**) Cell proliferation of TDSCs cultured in extracts of different scaffolds for 1, 4 and 7 days as determined by the CCK-8 assay. * indicates *P* < 0.05.

After 24 h of culture, the TDSCs were well attached to all the scaffolds and showed excellent viability, which confirmed that DCB, DCB-H and DCB-HO were nontoxic and suitable for cell growth (Figure 3A). The CCK-8 assay revealed a significantly reduced cell viability of TDSCs in the extracts from various scaffolds at both 4 and 7 days when compared to the normal culture medium (*P* < 0.05, Figure 3B). However, no significant differences in cell viability were observed among the different scaffold groups at the indicated time points (*P* > 0.05, Figure 3B).

### Osteogenic differentiation

**Figure 4.**
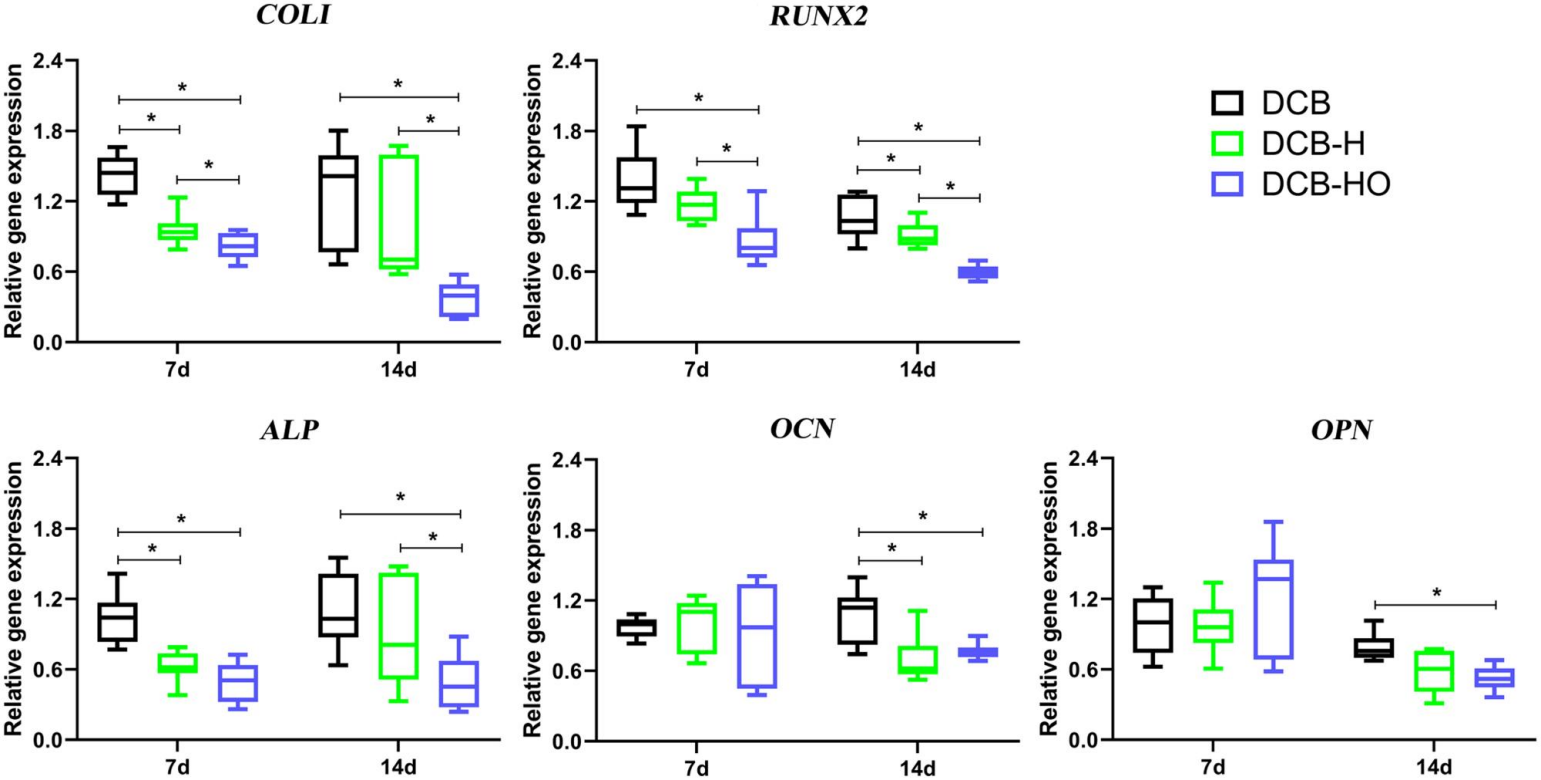
Osteogenic differentiation assays at the gene expression level. RT-PCR analysis of the relative gene expression of TDSCs cultured on the scaffolds for 7 and 14 days. The levels of gene expression were normalized to those of GAPDH. * indicates *P* < 0.05.

For TDSCs cultured on DCB, DCB-H and DCB-HO, RT-PCR analysis indicated that both heat treatment and H_2_O_2_ treatment reduced the osteoinductivity of DCB, whereas H_2_O_2_ treatment was more effective than heat treatment (Figure 4). At 7 days after culture, the expression levels of *COLI, RUNX2* and *ALP* in the DCB-HO group were significantly lower than those in the DCB group (*P* < 0.05). *COL1* and *ALP* expression in the DCB-H group was significantly lower than that in the DCB group (*P* < 0.05). In addition, the expression levels of *COLI* and *RUNX2* in the DCB-HO group were significantly lower than those in the DCB-H group (*P* < 0.05). At 14 days after culture, the expression levels of *COL1*, *RUNX2*, *ALP*, *OCN* and *OPN* in the DCB-HO group were significantly lower than those in the DCB group (*P* < 0.05). The expression levels of *RUNX2* and *OCN* in the DCB-H group were significantly lower than those in the DCB group (*P* < 0.05). In addition, the expression levels of *COL1*, *RUNX2* and *ALP* in the DCB-HO group were significantly lower than those in the DCB-H group (*P* < 0.05).

**Figure 5.**
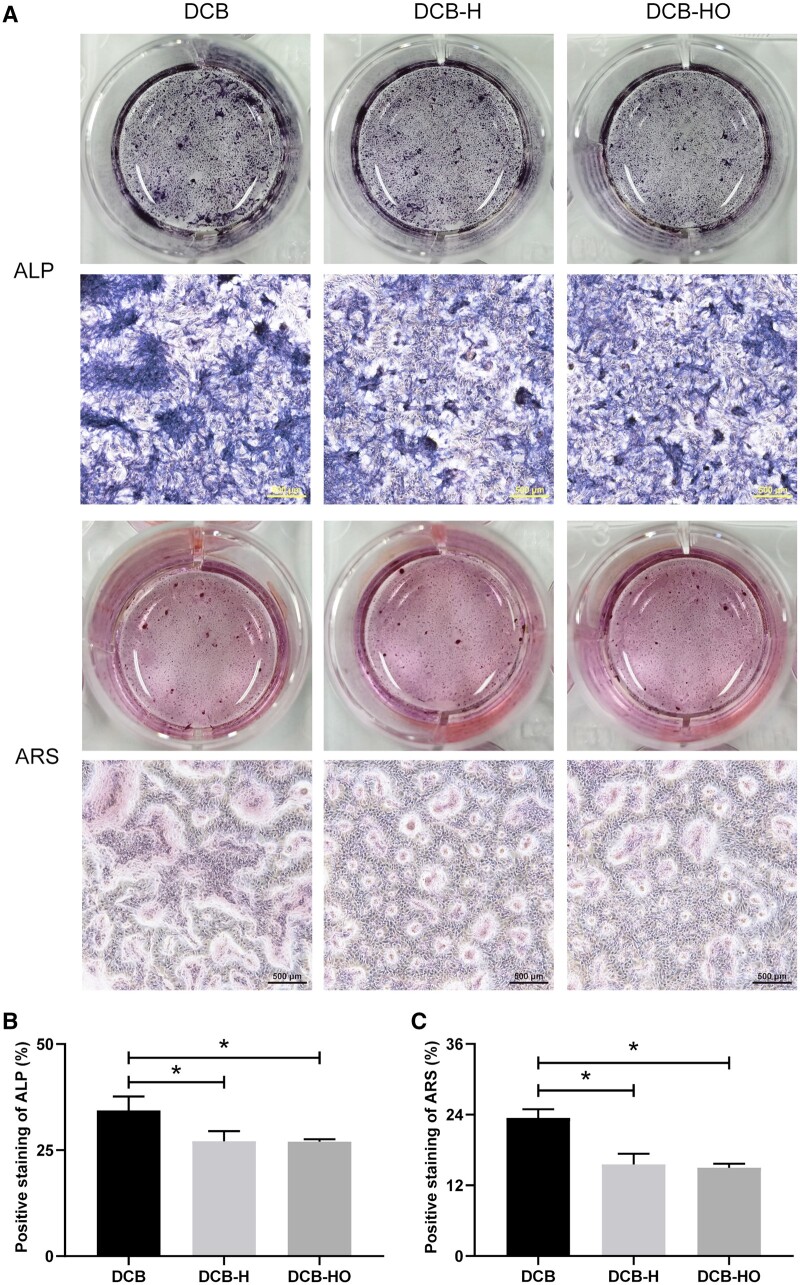
Osteogenic differentiation assays by specific staining. (**A**) ALP staining and ARS staining of TDSCs cultured on the scaffolds for 14 days. Scale bar = 500 μm. (**B**) Semiquantitative analysis of ALP-positive staining. (**C**) Semiquantitative analysis of ARS-positive cells. * indicates *P* < 0.05.

At the protein level, the expression of ALP and OPN at 7 days post-culture exhibited a decreasing trend in the DCB-H and DCB-HO groups; however, no significant differences were observed among the three groups (Supplementary Figure S2A and B). As anticipated, the expression of ALP at 14 days was significantly lower in the DCB-HO group than in the DCB group (*P* < 0.05). More significantly, the expression of OPN at 14 days was also significantly lower in the DCB-HO group than in both the DCB and DCB-H groups (*P* < 0.05, Supplementary Figure S2A and B). Accordingly, both ALP staining and ARS staining revealed significantly lower expression in the DCB-H and DCB-HO groups than in the DCB group under osteogenic induction conditions (*P* < 0.05, Figure 5), which also confirmed the RT-PCR data showing the expression of osteogenic gene markers.

### Macroscopic observations

**Figure 6.**
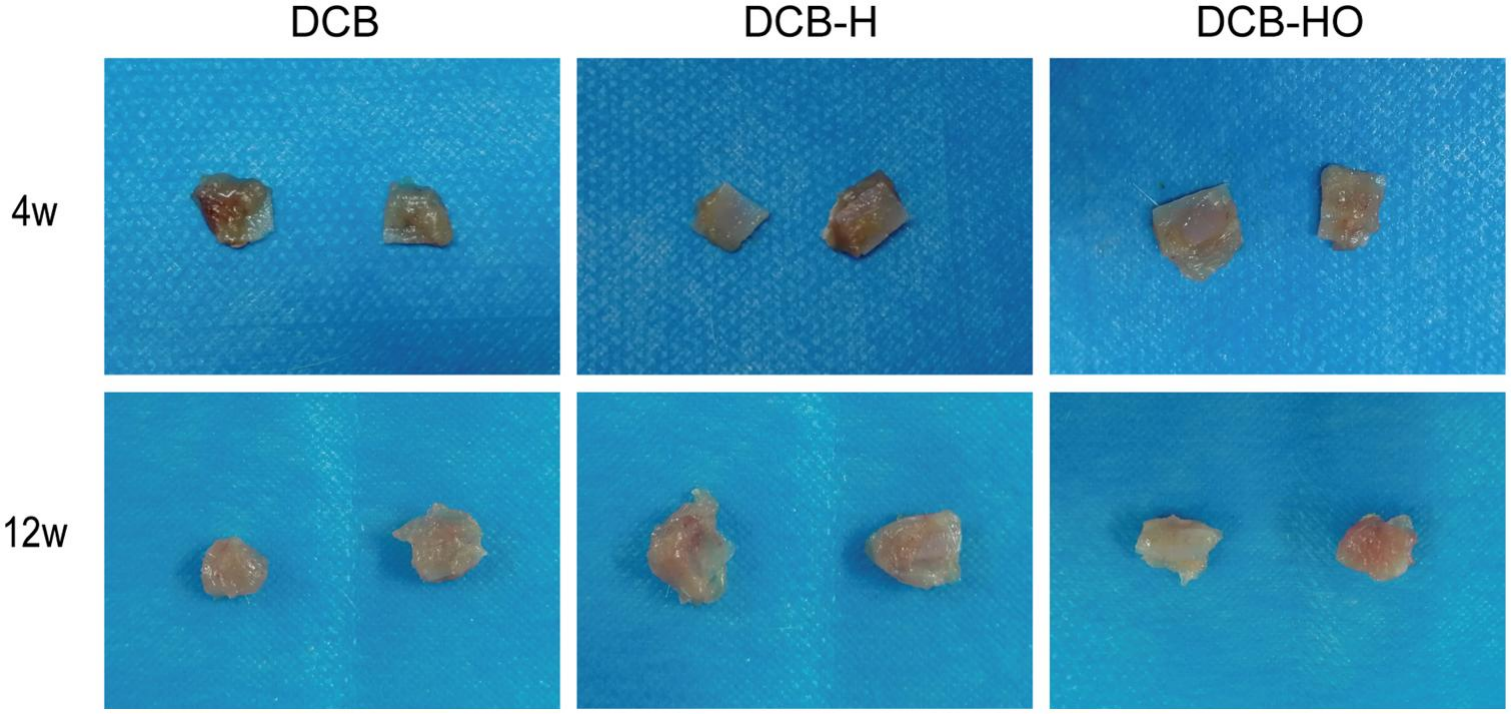
Gross observation of the implanted scaffolds at 4 and 12 weeks after surgery.

There was no evidence of infection at the surgical sites for any of the animals. No significant difference was found in the gross appearance of the implanted scaffolds during necropsy (Figure 6).

### Histological analysis

**Figure 7.**
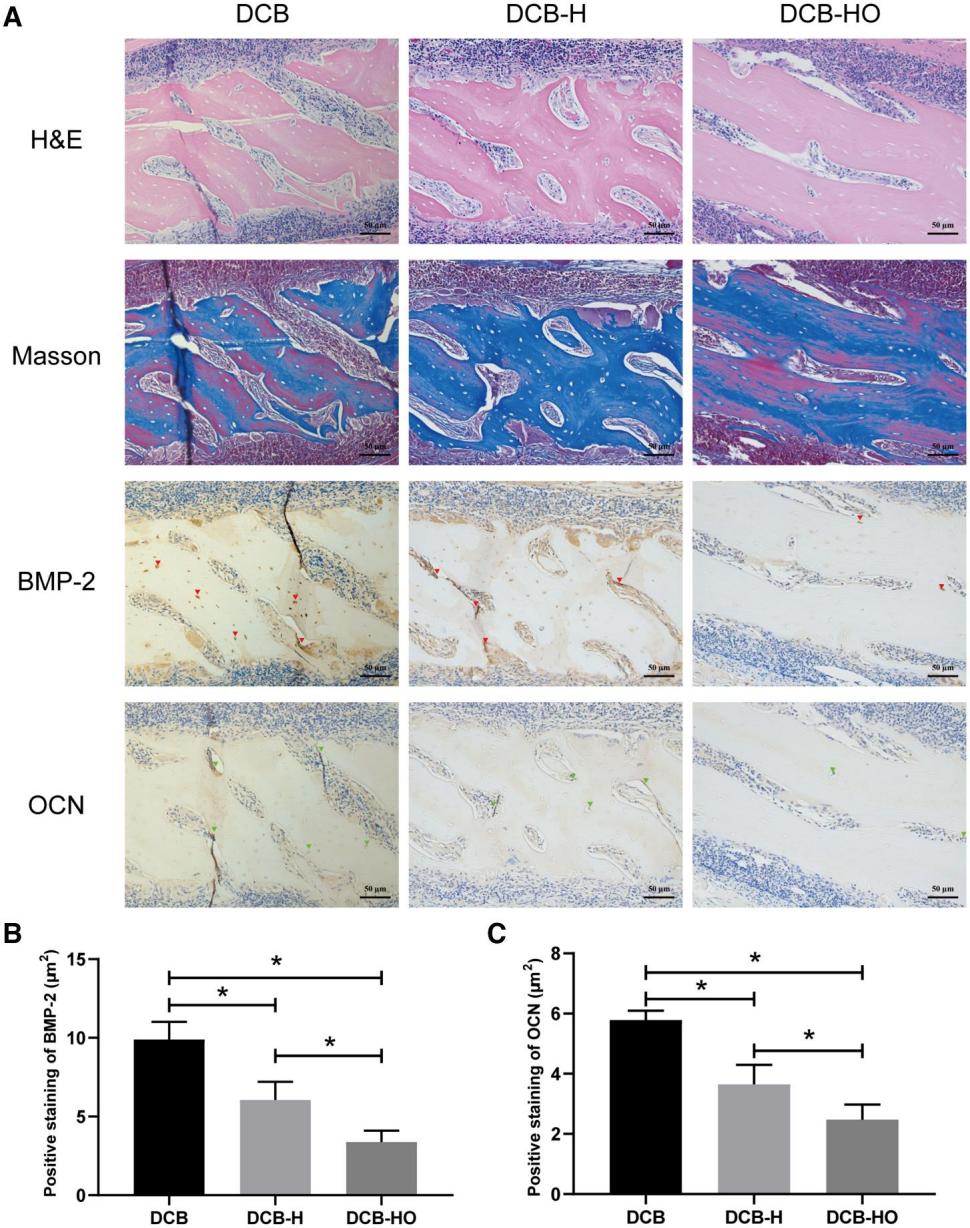
Histological analysis of SD rats implanted subcutaneously 4 weeks after surgery. (**A**) H&E, Masson and IHC staining of the regions implanted with the scaffolds. The red arrow indicates positive staining for BMP-2. The green arrow indicates positive staining for OCN. Scale bar = 50 μm. (**B**) Semiquantitative analysis of BMP-2-positive staining. (**C**) Semiquantitative analysis of OCN-positive staining. * indicates *P* < 0.05.

**Figure 8.**
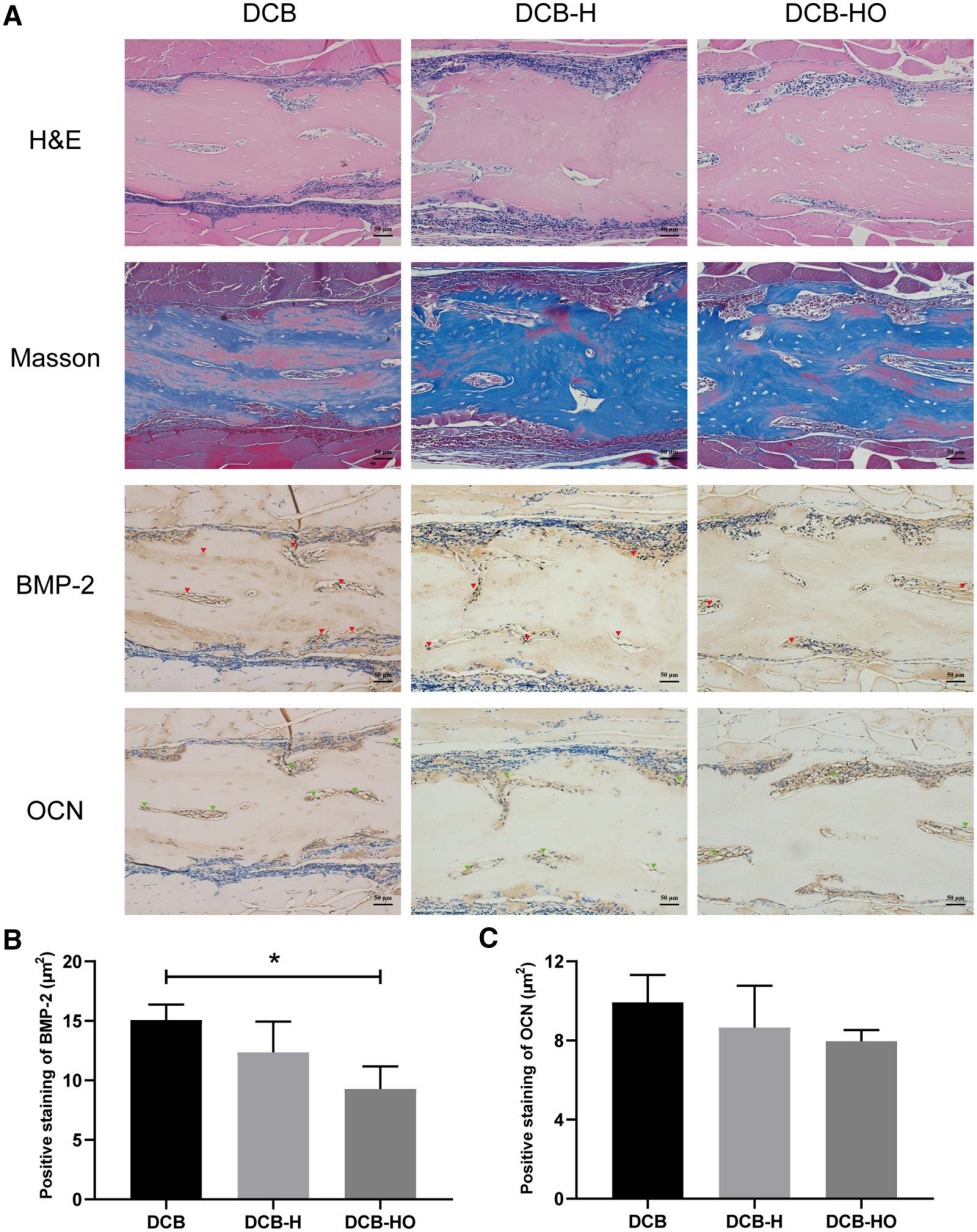
Histological analysis of SD rats implanted subcutaneously 12 weeks after surgery. (**A**) Representative images of H&E, Masson and IHC staining of the regions implanted with the scaffolds. The red arrow indicates positive staining for BMP-2. The green arrow indicates positive staining for OCN. Scale bar = 50 μm. (**B**) Semiquantitative analysis of BMP-2-positive staining. (**C**) Semiquantitative analysis of OCN-positive staining. * indicates *P* < 0.05.

**Figure 9.**
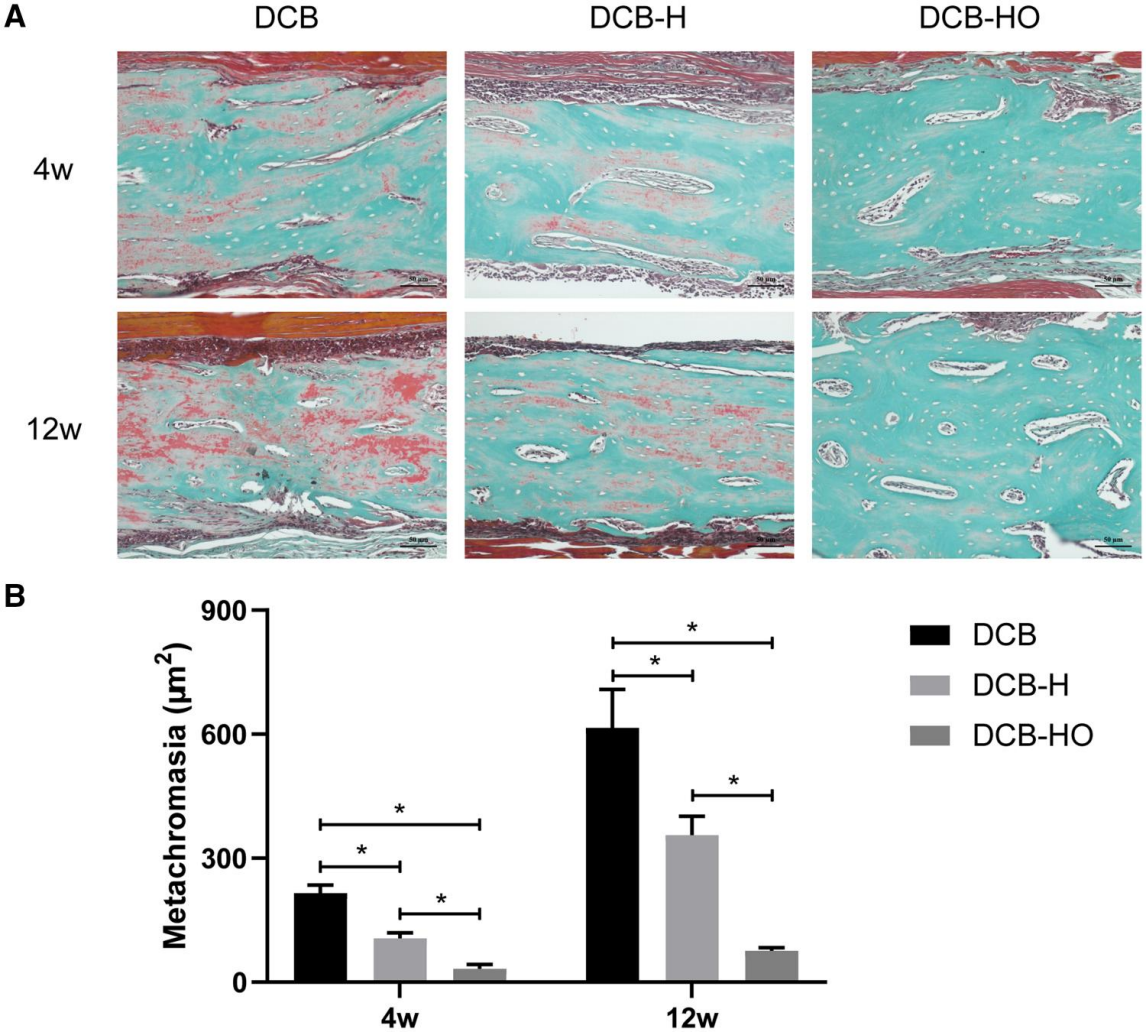
Histological analysis of neo-osteoid formation after subcutaneous implantation in SD rats for 4 and 12 weeks after surgery. (**A**) Representative images of Goldner trichrome staining of the regions implanted with the scaffolds. Scale bar = 50 μm. (**B**) Semiquantitative analysis of neo-osteoid formation (red area within the scaffolds). * indicates *P* < 0.05.

At 4 weeks after surgery, H&E and Masson staining revealed that the implanted scaffolds were encircled by a substantial infiltration of inflammatory cells (Figure 7A). There was more positive staining for BMP-2 and OCN in the DCB group than in the DCB-H and DCB-HO groups (BMP-2: DCB vs DCB-H vs DCB-HO = 9.89 ± 1.13 μm^2^ vs 6.06 ± 1.14 μm^2^ vs 3.38 ± 0.72 μm^2^; OCN: DCB vs DCB-H vs DCB-HO = 5.78 ± 0.31 μm^2^ vs 3.65 ± 0.64 μm^2^ vs 2.48 ± 0.50 μm^2^; Figure 7B and C). In addition, more BMP-2 and OCN-positive cells were detected in the DCB-H group than in the DCB-HO group (Figure 7B and C). At 12 weeks after surgery, a reduction in the number of inflammatory cells around the implanted scaffolds was observed by H&E and Masson staining (Figure 8A). BMP-2 staining was more strongly increased in the DCB group than in the DCB-HO group (DCB: 15.06 ± 1.30 μm^2^, DCB-HO: 9.28 ± 1.89 μm^2^; Figure 8B). However, there was no significant difference in BMP-2-positive staining between the DCB-H group and the DCB-HO group (DCB-H: 12.35 ± 2.59 μm^2^, DCB-HO: 9.28 ± 1.89 μm^2^; Figure 8B). In addition, there was no significant difference in the number of OCN-positive cells among the three groups (DCB: 9.92 ± 1.39 μm^2^, DCB-H: 8.66 ± 2.10 μm^2^, DCB-HO: 7.96 ± 0.56 μm^2^; Figure 8C). At 4 weeks after surgery, new osteoid formation was observed in the inner part of the implanted scaffolds (Figure 9A), and the semiquantitative results revealed that the area of metachromasia in the DCB group was greater than that in the DCB-H and DCB-HO groups (DCB: 215.89 ± 19.41 μm^2^, DCB-H: 106.14 ± 13.77 μm^2^, DCB-HO: 32.53 ± 10.40 μm^2^; Figure 9B). In addition, the area of metachromasia in the DCB-H group was larger than that in the DCB-HO group (Figure 9B). At 12 weeks after surgery, the area of osteoid formation became larger, and the semiquantitative results indicated that the DCB group had more osteoids than did the DCB-H and DCB-HO groups (DCB: 615.23 ± 92.85 μm^2^, DCB-H: 355.88 ± 45.38 μm^2^, DCB-HO: 106.26 ± 8.12 μm^2^; Figure 9B). Moreover, there were more osteoids in the DCB-H group than in the DCB-HO group (Figure 9B).

## Discussion

In this study, we successfully prepared DCB-H and DCB-HO for tendon/ligament repair through heat treatment and H_2_O_2_ treatment. The SEM, H&E and Masson staining results indicated that the microstructure of the scaffolds did not change after heat treatment or H_2_O_2_ treatment. In addition, the FT-IR results showed that DCB, DCB-H and DCB-HO were completely demineralized. The results of the IHC staining demonstrated that BMP-2 and OCN within the DCB were removed after heat treatment and H_2_O_2_ treatment. Moreover, H_2_O_2_ treatment was more effective than heat treatment. The results of the *in vitro* cell viability analysis demonstrated that DCB, DCB-H and DCB-HO had good biocompatibility. RT-PCR and WB analysis revealed that compared with DCBs, DCB-H and DCB-HO reduced the osteogenic differentiation of TDSCs, and compared with DCB-H, DCB-HO further reduced the osteogenic differentiation of TDSCs. ALP staining and ARS staining also revealed significantly lower expression in the DCB-H and DCB-H groups than in the DCB group under osteogenic induction conditions. According to the *in vivo* study, the histological staining results indicated that the scaffolds were surrounded by inflammatory cells, and the BMP-2- and OCN-positive areas were lower in the DCB-H and DCB-HO groups than in the DCB group at 4 weeks. At 12 weeks after surgery, the DCB-HO group had a smaller area of BMP-2-positive staining than did the DCB group. In addition, there were fewer the areas of osteoid formation in the DCB-H and DCB-HO groups than in the DCB group, and the DCB-HO group had the smallest area of osteoid formation among the three groups at 4 and 12 weeks after surgery. Our research indicated that heat treatment may diminish the osteoinductive properties of DCB, whereas treatment with H_2_O_2_ proved to be more efficacious in reducing osteoinduction compared to heat treatment.

As a material for repairing bone defects, DBM/DCB has attracted attention in the repair of tendons and ligaments, such as the ACL, MCL or patellar tendon [14–18]. Our previous study indicated that DCB possesses a well-preserved inherent structure of collagen, elemental biomechanics and good biocompatibility [29]. However, the osteoinductivity of DCBs needs to be addressed to reduce the occurrence of heterotopic ossification during soft tissue regeneration. H_2_O_2_ is a strong oxidant, that is used to eliminate the osteoinductivity of DCB. Previous studies demonstrated that H_2_O_2_-treated DCBs had a complete collagen structure and a remarkable reduction in osteogenesis, and the biomechanics (such as the elastic modulus and stiffness) of the DCB did not change significantly [26, 27, 30]. Our results also showed that H_2_O_2_ treatment could reduce the osteoinductivity of DCB. In addition to chemical treatment, heat treatment can also inactivate BMP-2 [28, 37]. We conducted this study because there was a lack of studies on the effects of heat treatment on the osteoinductivity of DCB. Although higher temperatures and longer wound durations completely inactivate BMP-2, the denaturation temperature of collagen is approximately 50–70°C, and higher temperatures are expected to cause significant damage [38, 39]. Thus, we chose this parameter (70°C for 8 h) for heat treatment according to published studies. To compare the effectiveness of the two treatments, we used the same processing time (8 h) for heat treatment and H_2_O_2_ treatment. Our study indicated that H_2_O_2_ treatment was more effective than heat treatment for improving osteoinductivity on the basis of the *in vitro* and *in vivo* results.

Although the mechanism of heterotopic ossification caused by implants is unclear, the inherent osteoinductivity of implants is one of the most important factors of heterotopic ossification. Previous studies have demonstrated that BMP-2 is the major factor leading to calcification [13, 40, 41]; therefore, BMP-2 was selected as an indicator for detecting alterations in the osteoinductivity of DCBs after heat treatment and H_2_O_2_ treatment. Transforming growth factor β1 (TGF-β1) and vascular endothelial growth factor (VEGF) can enhance calcification through synergistic activity with BMP-2 or through the coordination of angiogenesis and osteogenesis [42, 43]. However, our previous study revealed that VEGF and TGF-β1 within DCBs are removed after demineralization [30]. OCN is a factor expressed and secreted solely by osteoblasts and is partially stored in the bone matrix [44, 45]. OCN plays a vital role in bone formation and resorption, and it is essential for the alignment of biological apatites parallel to collagen fibrils, which could affect bone strength [46–49]. Thus, we chose the OCN concentration as the second indicator to evaluate the change in the osteoinductivity of DCBs. TDSCs exhibit self-renewal, clonogenicity and multipotency abilities, and they are derived from tendons [50]. TDSCs were selected to assess the osteoinductivity of DCB, DCB-H and DCB-HO *in vitro* because TDSCs might play a major role in tendon repair [51, 52]. Although reduced osteoinductivity of DCB-H and DCB-HO was identified *in vitro*, *in vivo* tests are still needed to verify the effects of heat treatment and H_2_O_2_ treatment on these scaffolds. Osteoids are unmineralized bone matrices that form prior to the maturation of bone tissues, and they initiate the process of forming new bone [53]. Therefore, osteoid staining was used to confirm our *in vivo* results.

However, there are several limitations in our study. First, our scaffolds were not tested for biomechanics, but heat treatment might affect the mechanical characteristics of DCBs. Second, only two biochemical components in the scaffolds were investigated, and other components of DCBs might lead to heterotopic ossification.

In conclusion, we successfully prepared DCB-H and DCB-HO through heat treatment and H_2_O_2_ treatment. Our study demonstrated that heat treatment could reduce the osteoinductivity of DCBs. In addition, H_2_O_2_ treatment was more effective than heat treatment. However, further studies are needed to confirm the potential of DCB-H and DCB-HO as biomaterials for tendon/ligament repair.

## Supplementary Material

rbae116_Supplementary_Data
