# Supplementary material for: The effects of heat and hydrogen peroxide treatment on the osteoinductivity of demineralized cortical bone: a potential method for preparing tendon/ligament repair scaffolds
Source: Regen Biomater. 2024 Sep 25;11:rbae116. doi: 10.1093/rb/rbae116 (PMC11471265; doi:10.1093/rb/rbae116)
Supplement: rbae116_Supplementary_Data [file rbae116_supplementary_data.docx]

Supplementary data


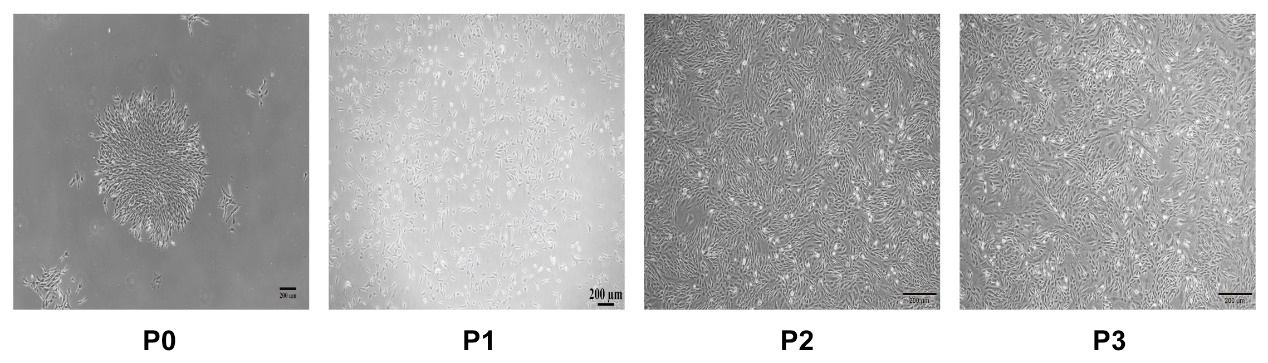


Fig. S1 Morphological features of TDSCs at different passages under normal culture medium.


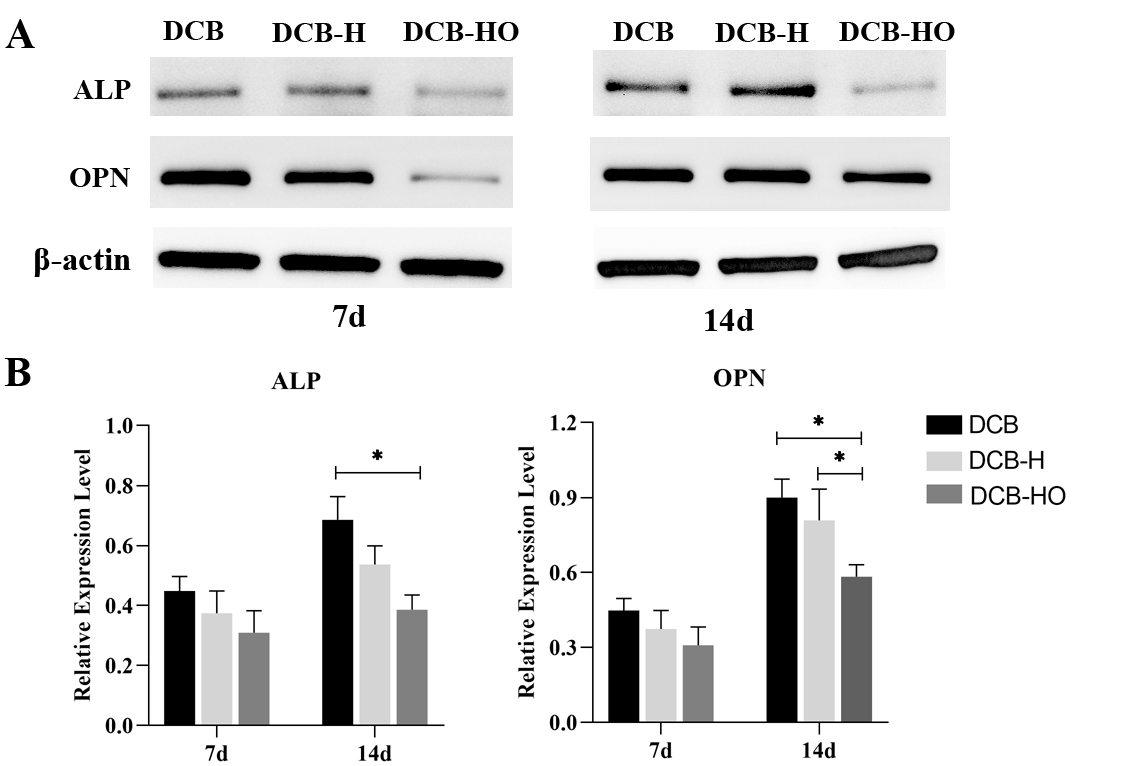


Fig. S2 Osteogenic differentiation assays at the protein expression level.

A: Representative western blot bands of TDSCs cultured on the different scaffolds at 7 and 14 days. B: Semi-quantitative analysis of tendon-specific proteins expression of TDSCs cultured on the different scaffolds at 7 and 14 days.

Data are normalized to β-actin. * indicates P<0.05.


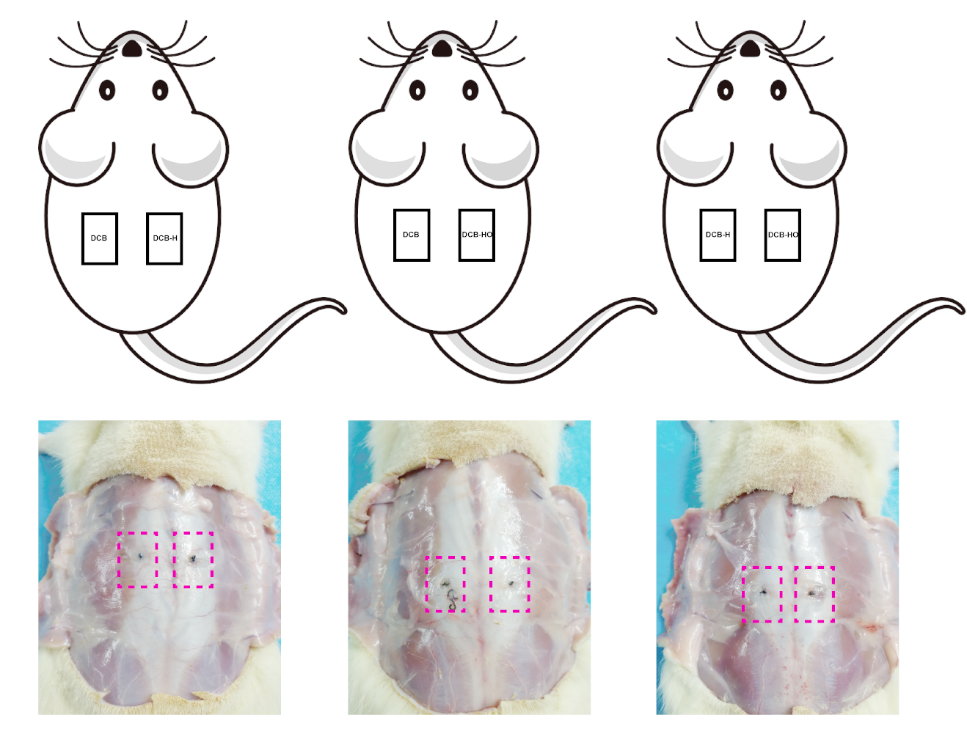


Fig. S3 The mode diagram and pictures of animals during surgery.
